# Dairy calf transportation in the United States: Challenges and strategies to improve animal welfare*

**DOI:** 10.3168/jdsc.2023-0452

**Published:** 2023-11-17

**Authors:** M.C. Cramer, J.A. Pempek, I.N. Román-Muñiz, L.N. Edwards-Callaway

**Affiliations:** 1 Department of Animal Sciences, Colorado State University, Fort Collins, CO 80523; 2 USDA-Agricultural Research Service Livestock Behavior Research Unit, West Lafayette, IN 47907

## Abstract

The objectives of this symposium review are to summarize relevant research and key welfare issues relative to calf transportation and identify strategies to mitigate welfare challenges. An important animal welfare concern across the US dairy industry is the transportation of preweaning calves from the source dairy to a calf-raising facility (e.g., calf ranches, heifer raising facilities, veal operations), auction, livestock market, or directly to slaughter. Millions of calves are transported annually in the United States and calf transport has garnered increased attention. Transportation stressors include limited (if any) access to food and water, commingling, environmental temperature changes, and a variety of handling techniques. Calves in the United States are often transported at an average age of 3 d, and in many cases, less than 24 h of age. Neonates are particularly vulnerable to transportation stressors due to their decreased ability to thermoregulate, underdeveloped immune system, and immature physiologic stress responses. In addition to age, fitness for transport is a key welfare consideration; recent data from the United States demonstrate that some source dairies transport compromised calves (i.e., dehydration, diarrhea, navel inflammation, and so on), leading to important welfare challenges during transportation. Calves arriving at US veal facilities have been reported to be dehydrated, lethargic, hypoglycemic, and may also have poor body condition, navel inflammation, and diarrhea. Thus, there is ample opportunity to target decision-making and producer-focused education not only at the source dairy, but also at each stage of transportation to address critical welfare concerns. In addition, the supply chain and procurement model that influence calf transport practices should be evaluated to determine potential opportunities to improve calf welfare. Here, we provide 5 potential strategies to improve the welfare of transported calves: (1) provide excellent newborn care that “preconditions” calves for transport, (2) assess calves' fitness-for-transport to ensure they can withstand the journey, (3) handle calves with care, (4) wait until calves are older to transport, and (5) reduce transport duration.

## Graphical Abstract

**Figure:**
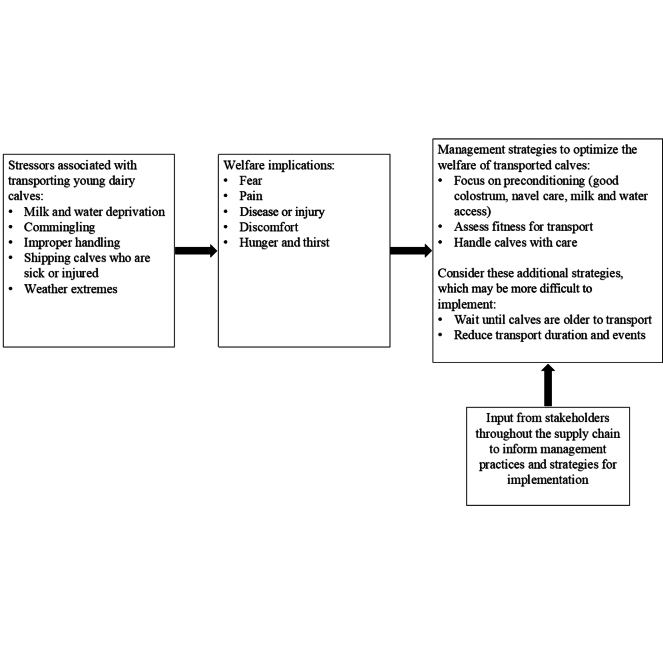

The transport of young dairy calves in the United States and Canada has recently garnered increased attention (Alley et al., 2020; Creutzinger et al., 2021; Roadknight et al., 2021). Calf transport is of particular concern because the neonatal calf is more susceptible to stressors associated with transport (e.g., feed and water restriction, commingling, various handling techniques, and thermal stress; Trunkfield and Broom, 1990) due to physiological responses to stress and immune systems that are both underdeveloped (Pardon et al., 2015; Hulbert and Moisá, 2016). Transported calves can be broadly classified into replacements and nonreplacements. Replacement heifers are female calves who will most likely enter a milking herd, whereas nonreplacements (sometimes referred to as surplus calves) are male and female calves who will not be retained for eventual entry into the milking herd, but instead will enter the veal or beef supply chains. This distinction is important because management practices often differ between replacements and nonreplacements, especially dairy bull calves (Shivley et al., 2019; Creutzinger et al., 2021, 2022). Approximately 43% of replacement heifer calves were sold or raised off-site with retained ownership, with approximately 15% of operations transporting calves more than 80 km (USDA, 2016). Given that neonatal calves have underdeveloped immune systems (Hulbert and Moisá, 2016), it is important to note that replacement calves leave dairies at an average age of 3 d (USDA, 2016). Nonreplacement calves are typically sold from the dairy farm at <1 wk of age in the United States (Shivley et al., 2019), and recent data show that over 60% are transported at <24 h of age (Cramer et al., 2024). Thus, a large number of calves are being transported off of US dairies at ≤1 wk of age, and often less than 24 h of age.

One critical aspect of pretransport calf management is colostrum management. Consensus recommendations for transfer of passive immunity (**TPI**) suggest 10% or less of calves should have serum IgG concentrations of <10 g/L or poor TPI (Lombard et al., 2020). However, England et al. (2023) reported 23.4% of bob veal calves had poor TPI on arrival at a slaughter establishment in the Midwestern United States, and similar prevalence estimates have been reported for formula-fed veal (Pempek et al., 2017; Renaud et al., 2018a) and dairy-beef calves (Pempek et al., 2023). There is growing evidence that colostrum management practices sometimes differ for replacement compared with nonreplacement calves, with nonreplacement calves sometimes receiving inadequate colostrum (Shivley et al., 2019; Creutzinger et al., 2022). However, both replacement and nonreplacement dairy calves are at high risk of disease in early life (Timmerman et al., 2005; Pardon et al., 2015). For example, previous research at a commercial veal calf-raising facility in Ontario, Canada, documented 7.5% of calves died within the first 11 wk of the production period; almost 90% of calves were treated with antimicrobials at least once for diarrhea; and approximately 90% of calves were treated once or more for respiratory disease (Scott et al., 2019; Goetz et al., 2021). Thus, good colostrum management on the dairy is paramount to reduce calves' risk of morbidity and early mortality after transport.

“Fitness for transport,” referred to as an animal's ability to withstand transportation without compromising welfare, is another aspect of pretransport management that can have important implications for calf welfare (Edwards-Callaway et al., 2019). Recently, research has examined this topic in calves. A Canadian study identified that 37% of calves had at least one abnormal health condition (e.g., depressed behavioral attitude score, fever, respiratory disease, diarrhea, and navel inflammation) before being transported (Wilson et al., 2020a). A study in the western United States found that nearly half of transported calves had at least one health abnormality (e.g., diarrhea, dehydration, joint inflammation, navel inflammation) before transport (Cramer et al., 2024). Transporting sick or injured calves (i.e., “compromised” calves) is a serious welfare concern because the stressors are compounded for compromised calves and existing conditions (i.e., disease, dehydration, and so on) can worsen during transport. Furthermore, calves who are sick upon arrival to calf-rearing facilities are at a greater risk for death and poor growth compared with calves who arrive in good condition (Renaud et al., 2018a,b).

During transport and marketing, calves may be exposed to a variety of factors that can result in welfare concerns if not properly managed; these include improper handling resulting in stress and fear, lack of bedding resulting in discomfort or potential injury, increased exposure to pathogens that can increase disease risk, commingling resulting in stress or fear, weather extremes resulting in discomfort, lack of available milk or water, dehydration, and thermal stress (reviewed by Roadknight et al., 2021). One important welfare concern centers around the withholding of milk and water (reviewed by Roadknight et al., 2021). In the United States, milk or water (or both) are typically not provided to calves during marketing or transport (reviewed by (Creutzinger et al., 2021), and calves can be transported for up to 28 consecutive hours per federal regulations in the United States (United States Code, 1994). These factors may explain why a large proportion of calves are hypoglycemic and dehydrated after transport (Pempek et al., 2017; Wilson et al., 2020b; England et al., 2023). In addition to the biological implications of fasting, the impact of withholding milk and water on calves' affective state should also be considered, as it is likely that milk and water deprivation induce feelings of hunger and thirst (reviewed by Roadknight et al., 2021). Additionally, indirect marketing can increase their time in transit and exposure to handling and commingling events. According to the most recent national estimates, approximately three-fourths of nonreplacement male and 8.5% of nonreplacement female calves were marketed indirectly in 2014 (USDA, 2016). The stressors that calves may experience during marketing include exposure to new environments and animals, a variety of handling techniques, and commingling (reviewed by Roadknight et al., 2021). Compromised calves are also a challenge for livestock auctions or markets to handle and including these stakeholders in decision-making is critical.

Varying degrees of compromised welfare have been reported in young calves on arrival at their destination (Pempek et al., 2017; Renaud et al., 2018a; England et al., 2023). For example, the most common physical health concern reported by England et al. (2023) on calf arrival at a slaughter establishment in the Midwestern United States was dehydration (mean: 68.6%), followed by thin body condition (39.8%) and navel inflammation (25.7%). Approximately 73.4% of calves were also considered hypoglycemic (blood glucose concentration <4.95 mmol/L; Renaud et al., 2022) in this study, and the odds of hypoglycemia were 3 times greater for male nonreplacement calves compared with female nonreplacement calves (England et al., 2023). Pempek et al. (2017) found that calves had diarrhea (14%), respiratory disease (0.5%), depressed attitudes (14%), inflamed navels (27%), and dehydration (35%) at arrival to veal facilities in Ohio. A 2018 Canadian study collected health measurements on calves at arrival to a veal facility and found that approximately 46% of calves arrived at the facility with some level of dehydration (Renaud et al., 2018b). Additionally, 32.7% and 18% of calves arrived with no subcutaneous fat covering and were emaciated, respectively (Renaud et al., 2018b). Furthermore, nearly 30% of calves had clinical signs of navel infection (Renaud et al., 2018b). While information is lacking to fully understand at which point in the transport process calf condition deteriorates, recent work suggests that it is a combination of poor decision-making on the dairy farm to ship compromised calves (Wilson et al., 2020a; Cramer et al., 2024) and exposure to the stressors of transportation (Roadknight et al., 2021) that lead to a large proportion of calves arriving at auction markets in poor condition (Wilson et al., 2020b). Clearly, calves experience welfare challenges during transportation. Next, we offer 5 potential strategies to improve the welfare of transported calves.

First, the dairy industry should focus on preconditioning practices for calves before transport. Preconditioning is a term often used in the beef sector and is a set of management practices intended to prepare the animal for their next phase of production (Hilton, 2015). This concept can be useful to consider on the dairy; what management practices can we implement to help the calf be successful during and after transport? Of primary concern for transported calves are practices that can prevent failed transfer of passive immunity (**FTPI**), dehydration, navel inflammation, and hypoglycemia. First and foremost, every calf should receive at least 150 to 200 g of IgG within 2 h of birth (Godden et al., 2019). This will likely reduce the proportion of calves identified with FTPI after transport and ensure calves have a reduced risk of disease (Godden et al., 2019). Next, given the large proportion of calves identified with navel inflammation (Pempek et al., 2017; England et al., 2023), navel antisepsis shortly after birth and maintaining a clean calving pen are considered best practices (Mee, 2008). However, more work is needed to identify effective methods to prevent navel infections, as indicated by a recent study that found a 7% iodine tincture did not prevent infection (Van Camp et al., 2022). Finally, calves should be fed a milk meal as close to transport as possible to prevent dehydration and hypoglycemia. Ideally, milk and water would be available at all times throughout the transport process; however, this is not common due to the logistic challenges of feeding calves milk or providing water while in transit (Creutzinger et al., 2021). Calves spend the majority of the time in transit lying down (Marcato et al., 2020; Cramer et al., 2024); however, it is unknown if they will stand to drink water or milk during transit, or if milk and water are provided during marketing; these aspects should be investigated further.

Second, calves should be assessed for fitness for transport at each point in the supply chain. Although no regulations exist regarding the transport of compromised cattle in the United States, industry recommendations from Calf Care Quality Assurance (**CCQA**) and the American Association of Bovine Practitioners (**AABP**) state that sick or injured calves should not be transported unless animals are transported directly to receive veterinary care (AABP, 2019; CCQA, 2022). Calf condition should be assessed at each point in the supply chain (i.e., at the source dairy before transport, during transportation, during marketing, and upon arrival). More specifically, calves with disease (i.e., diarrhea, navel or joint inflammation, or respiratory disease), lethargy, dehydration, bone fractures, dyspnea, thin body condition, severe lames, or open wounds or calves that are unable to stand and walk easily should not be transported and should receive prompt treatment (CCQA, 2022). If the animal cannot be appropriately treated or the prognosis is poor, euthanasia must be considered. Also, facilities that allow for calves to be treated or rest may be lacking at some points in the supply chain, which may make treating calves more difficult. Transporting calves with these conditions can exacerbate existing pain or discomfort and could cause calf condition to deteriorate. Implementing protocols on-farm that include an evaluation of fitness to transport before shipment could be practical, easily implemented, and beneficial for the improvement of calf condition upon arrival at the next destination in the supply chain.

Third, calves should be handled with care, both from a human-animal interaction standpoint, but also ensuring the trailer environment is suitable for calves. Calves can be somewhat difficult to handle for people who are not used to doing so because they often move slowly, may be unsteady on their feet, and do not respond to being moved via flight zones (AABP, 2019). Based on the authors' experiences, we suggest the following handling techniques for calves and have described them previously (Pempek and Cramer, 2023). Briefly, calves should be moved individually using 3 potential techniques: (1) using 2 hands and standing beside the calf, place one hand under the chin and one hand around the rump; apply gentle pressure to the rump and guide the calf's head with the other hand; (2) encourage the calf to walk forward by running a hand gently up their spine from their tail to their head; and (3) pick calves up by placing one arm under the chest to support the head and chest and one arm around the rump up against the back legs, using care not to hurt the tail. An electric prod should never be used for calves and they should not be dragged, thrown, kicked, or handled solely via the ears or tail (AABP, 2019). The trailer environment can also be adjusted to facilitate calf comfort. The thermoneutral zone for young calves is between 15 and 26°C (Spain and Spiers, 1996; Davis and Drackley, 1998), and depending on the season and time of day, ambient temperatures and conditions in the trailer can differ drastically. Calves cannot easily thermoregulate (Davis and Drackley, 1998), so attention must be given to providing environmental factors that help the calf maintain a normal body temperature. Using proper handling techniques and adjusting the trailer environments could be easy to implement on-farm and would only require minimal changes to existing management practices.

Last, we propose 2 strategies that may be difficult to implement immediately but should be further investigated to optimize calf welfare during transport. The first strategy we propose is to retain calves on the source dairy longer (Pempek and Cramer, 2023). Calves in the United States are often transported at less than 24 h of age, but this may be detrimental to their welfare and there are potential benefits to transporting calves at an older age; for example, recent work in Canada suggests that transporting calves when they are at least 7 d of age results in greater ADG and reduced odds of disease after transport (Goetz et al., 2023a). Next, reducing transport duration and the number of events may help improve welfare by reducing the overall time calves are exposed to stressors and withheld from milk and water (Pempek and Cramer, 2023). Shorter transport durations have been associated with reduced mortality, normal blood glucose concentrations, increased BW, and improved health posttransport (Boulton et al., 2020; Rot et al., 2022; Goetz et al., 2023a,b). Although reducing transport duration may not be easy, strategies to shorten transport duration (i.e., minimizing the number of stops in a route to pick up more calves) and adding rest stops for calves should be investigated further. For example, calves transported 6 h versus 16 h had reduced incidence of dehydration in the 3 d following transport, greater ADG in the 50 d posttransport, and decreased incidence of diarrhea in the 14 d following transport (Goetz et al., 2023a). Calves who are marketed indirectly and therefore travel through one or more auctions or livestock dealers are exposed to more stressors and for a longer duration (i.e., multiple loading and unloading events, longer time off of milk and water, commingling, and so on; Pempek et al., 2017; Creutzinger et al., 2021). Working with supply chain stakeholders to discuss the feasibility of extending the time calves stay on the dairy and reducing transport duration and events are important next steps to identify strategies that can not only positively affect welfare but are also realistic.

Some strategies (e.g., colostrum, water and milk feeding, and navel disinfection) will likely be easier to implement than other strategies (e.g., retaining calves on the dairy for longer and reducing transport duration and events). The procurement model for calves, especially nonreplacements, does not always lend itself to making changes that optimize calf welfare. The procurement model is a reality of the current system unlikely to change soon; however, building accountability into the supply chain may help. To the authors' knowledge, there are no widespread accountability efforts built into the supply chain (i.e., a dairy can ship compromised calves without penalty, and an auction can sell compromised calves without penalty). Another option is for calf-raising facilities is to require dairies to perform certain preconditioning practices (i.e., colostrum feeding, navel antisepsis, and so on) for purchased calves; measurable outcomes (e.g., serum total proteins, BW, and health) can also be collected at the calf-raising facilities to verify practices. We encourage dairy stakeholders to identify strategies to increase accountability throughout the supply chain and suggest more research to understand which factors are most beneficial for improving calf condition and are feasible to implement.

Improving the welfare of transported calves throughout the supply chain will require action from all individuals at each point along this supply chain; therefore, it is critical that these people are involved with developing solutions. Bolton and Von Keyserlingk (2021) suggest that strategies for managing nonreplacement calves, including transportation, should involve the voices of all stakeholders, including the public, from the onset (i.e., from identifying research question through and including dissemination of research and industry recommendations). Thus, the authors suggest that strategies to improve calf welfare during transportation include input from all sectors of the industry (i.e., dairy farmers, employees on calf-raising facilities, transport drivers, auction and livestock market employees, scientists, veterinarians, and consumers) to improve the welfare of transported calves. Although it is unlikely that only changing one management practice will lead to the systemic change that is needed to improve the welfare of transported calves, simple changes taken by individuals across the supply chain will promote progress in this important space.
